# Perception of Corporate Hypocrisy in China: The Roles of Corporate Social Responsibility Implementation and Communication

**DOI:** 10.3389/fpsyg.2020.00595

**Published:** 2020-04-22

**Authors:** Yiqi Zhao, Yuanjian Qin, Xianfeng Zhao, Xiao Wang, Leilei Shi

**Affiliations:** ^1^School of Management, Wuhan University of Technology, Wuhan, China; ^2^Business School, Hebei GEO University, Shijiazhuang, China; ^3^International Business School Suzhou, Xi’an Jiaotong-Liverpool University, Suzhou, China; ^4^State Grid Hebei Electric Power Company Xingtai Power Supply Branch, Xingtai, China

**Keywords:** corporate social responsibility, CSR motivation perception, corporate hypocrisy, communication, implementation

## Abstract

In the past two decades, corporate hypocrisy has become a phenomenon that cannot be ignored in Corporate Social Responsibility (CSR) practice (Wagner et al., 2009) and has thus become a concern for management scholars (Cho and Lee, 2019). Using smartPLS, based on attribution theory, this paper takes 28 Chinese listed enterprises as examples to explore the influence of CSR motivation on its communication and implementation, as well as the impact of CSR implementation and promotion on consumers’ perception of corporate hypocrisy. The research finds a negative correlation between value-driven motivation and corporate hypocrisy and a positive correlation of performance-driven motivation and stakeholder-driven motivation with corporate hypocrisy. The theoretical contribution of this paper is mainly reflected in the following four aspects. (1) It describes the scale of CSR implementation research and enriches the measurement tools of CSR implementation. (2) It enriches and expands research results in the field of CSR motivation perception. From the perspective of CSR and attribution theory, this study explores the influence of consumers’ perception of CSR motivation on CSR communication and CSR implementation. (3) It supplements research results in the field of corporate hypocrisy. The influence of CSR communication and CSR implementation on corporate hypocrisy is clarified. (4) It clarifies the impact of CSR communication on CSR implementation so as to help enterprises better match CSR communication strategy and CSR implementation in practice and reduce consumers’ perception of corporate hypocrisy. It is suggested that enterprises find their own positioning on CSR motivation, which provides a reference with which enterprises can make better decisions on CSR communication strategy after implementing CSR behavior and provides empirical evidence for the research on CSR motivation perception and corporate hypocrisy in China.

## Introduction

Corporate social responsibility (CSR) is a different kind of responsibility to legal and economic responsibilities; the goal of CSR is to pursue the enterprise’s business activities whilst having a positive impact on social development (McWilliams and Siegel, 2001). However, there is generally a gap between the commitment made by enterprises to social responsibility and their actual behavior (Jahdi and Acikdilli, 2009). Scholars describe such gaps in enterprises’ fulfillment of their commitments to social responsibilities as “corporate hypocrisy.” In the past two decades, corporate hypocrisy has become a phenomenon that cannot be ignored in CSR practice (Wagner et al., 2009) and has thus attracted the attention of management scholars (Cho and Lee, 2019). At present, scholars mainly study the conceptual definition, behavioral classification, motivational analysis, and implementation strategy of corporate hypocrisy from different perspectives such as social psychology, marketing, strategic management, and organizational behavior (Ju et al., 2014). Based on CSR, Wagner et al. (2009) and Fassin and Buelens (2011) used qualitative research to explore the leading variables of corporate hypocrisy behavior.

Skarmeas and Leonidou (2013) found that extrinsic motivation (referred to as egoism and stakeholder-driven motivation in their paper, based on Ellen et al., 2006) helps lead consumers to doubt enterprises’ commitments to CSR, whereas intrinsic motivation (referred to as value-driven motivation) restrains doubt about enterprises’ commitments to CSR. Wagner et al. (2009) defined “corporate hypocrisy” as the phenomenon of CSR communication that is inconsistent with actual action in the process of CSR. Morsing (2017) found that CSR communication seems to be a “double-edged sword” that may arouse the suspicion of stakeholders. However, it can also indirectly stimulate CSR action, thus becoming an effective driving force for CSR implementation. The question arises of how to make use of the positive effect of CSR and communication to make it an effective driving force for CSR. Among the existing studies, the literature exploring the impact of CSR and communication on CSR implementation is scarce (Ihlen et al., 2011). In previous studies, the research on CSR implementation is mostly qualitative, with empirical methods seldom used (Fassin and Buelens, 2011). In the existing literature, there are few studies exploring the impact of consumer CSR motivation perception on CSR communication and implementation. Based on these questions and on attribution theory, this study explores the relationship between perceived CSR motivation and CSR implementation and communication and the impact of CSR implementation and communication on consumers’ perceptions of corporate hypocrisy.

Since real motivation is embedded in the decision-making process of enterprises and cannot be directly measured and evaluated, this paper attempts to study the customer perception and evaluation of CSR activities from the perspective of public perception and attribution. It measures CSR motivation and corporate hypocrisy through consumer perception.

The conclusion is as follows: there is a negative correlation between value-driven motivation and corporate hypocrisy, and stakeholder-driven motivation is positively correlated with corporate hypocrisy. Value-driven motivation is negatively correlated with corporate communication, while performance-driven motivation is positively correlated with corporate communication. Furthermore, there is a positive correlation between CSR communication and CSR implementation, and CSR communication is positively correlated with corporate hypocrisy.

## Literature Review

### Perception of CSR Motivation

Attribution theory explains consumers’ perceived attribution of CSR motivation and how this cognitive perception affects their subsequent attitudes and behaviors (Kelley and Michela, 1980). Consumers mainly attribute CSR to two types of motivation: extrinsic motivation and intrinsic motivation. Extrinsic motivation is there CSR is seen as an attempt by enterprises to use socially responsible behaviors to increase profits or to deal with pressure from stakeholders (such as shareholders, society, environment, etc.), while intrinsic motivation is where CSR is seen as a true attempt by enterprises to solve social problems and improve overall social welfare through socially responsible behaviors (Romani et al., 2016). Consumers attribute motivation to the behavior of the company, and these attributions influence their subsequent responses to the company (Campbell and Kirmani, 2000). Skarmeas and Leonidou (2013) used attribution theory (Kelley and Michela, 1980) and found that extrinsic motivation (in Skarmeas and Leonidou’s, 2013, paper, referred to as egoism and stakeholder-driven motivation, based on Ellen et al., 2006) is positively associated with consumers’ doubts about enterprises’ commitments to CSR, whereas intrinsic motivation (referred to as value-driven motivation) is negatively related to consumers’ doubts about enterprises’ commitments to CSR.

#### Intrinsic Motivation

Romani and Grappi (2014) proposed the concept of consumers’ perception of enterprises as having an internal motivation for CSR. Internal CSR motivation perception refers to consumers’ beliefs that the motivation of enterprises to engage in CSR activities comes from the enterprises’ own moral standards and social missions and that these activities are not tools used by enterprises to pursue commercial interests. Examples of such activities include manufacturing green products, paying attention to green development, and carrying out green CSR activities, expressions of interest in matters such as the improvement of the environment and the sustainable development of society, and green policies and environmental protection initiatives (Romani et al., 2016). The perception that CSR activities are internally motivated will affect consumers’ evaluation of those CSR activities, and, in consequence, will affect consumers’ responses (Becker-Olsen et al., 2006). Consumers that perceive a high level of intrinsic motivation will form positive evaluations of enterprises’ CSR activities, which will lead to increased purchase intention, whereas consumers that perceive a low level of intrinsic motivation will form negative evaluations of enterprises’ CSR activities, which will lead to lower purchase intention (Kang et al., 2012).

Intrinsic motivation refers to enterprises performing their CSR activities based on a moral perspective, in order to improve overall social welfare. When consumers attribute CSR behavior to intrinsic motivation, they will perceive that enterprises genuinely desire to solve social problems and will therefore have respect for the enterprise (Skarmeas and Leonidou, 2013). Value-driven motivation refers to enterprises engaging in CSR behavior because of their moral, ethical, and social ideals and standards (Ellen et al., 2000). Consumers who perceive enterprises to have value-driven motivation for their CSR activities believe that enterprises genuinely care about social problems and desire to solve social problems by means of their socially responsible behavior and improve the social value of the enterprise. When consumers attribute CSR behavior to value-driven motivation, they feel that CSR practices are sincere (Becker-Olsen et al., 2006), and their suspicion of corporate hypocrisy will be weak (Skarmeas and Leonidou, 2013). We therefore formulate the following hypothesis:

H1: Intrinsic motivation (value-driven motivation) is negatively associated with corporate hypocrisy.

#### Extrinsic Motivation

Extrinsic motivation refers to enterprises’ desire to use CSR activities to achieve their own commercial objectives. Consumers’ perception of an extrinsic motivation for enterprises’ CSR activities will lead to the consumers feeling cheated, resulting in negative reactions (Du et al., 2010).

Motivation based on self-interest (or performance) refers to the motivation to maximize profits or achieve other commercial objectives by making use of CSR behavior (Ellen et al., 2006). If consumers believe that the reason enterprises engage in CSR behavior is to opportunistically pursue their own interests, they will believe that enterprises are manipulating and misleading consumers (Skarmeas and Leonidou, 2013). Consumers believe that it is immoral to be performance-driven and for enterprises to regard the fulfillment of social responsibilities as a means to obtain commercial benefits (Forehand and Grier, 2003; Vlachos et al., 2009). When consumers attribute enterprises’ CSR activities to the pursuit of commercial performance objectives, they may suspect the presence of corporate hypocrisy. We therefore formulate the following hypothesis:

H2: Self-interest-driven motivation (based on commercial performance) is positively associated with corporate hypocrisy.

Stakeholder-driven motivation refers to enterprises’ motivation to participate in CSR in order to meet the expectations of their various stakeholders (Vlachos et al., 2009). In stakeholder-driven motivation perception, consumers attribute enterprises’ CSR behavior to the motivations of stakeholders, which means that the enterprises take action to deal with the pressure from stakeholders such as shareholders, employees, customers, suppliers, and society as a whole (Skarmeas and Leonidou, 2013). This attribution has a negative effect on the perceived sincerity of the enterprises when they engage in CSR behavior, because consumers believe that enterprises only implement CSR behavior in response to stakeholder pressure and would not do so in the absence of such pressure (Smith and Hunt, 1978). Enterprises may engage in social responsibility activities in order to obtain rewards or avoid punishment from stakeholders (Ellen et al., 2000; Vlachos et al., 2009). For example, in order to complete its annual report, an enterprise may have to report on the activities related to social responsibility that it engaged in during the year; in consequence of being punished for violation of regulations, in order to repair damage to their image and regain the trust of consumers and investors, they may engage in social responsibility activities. In such a context, stakeholder-driven motivation attribution may cause consumers to suspect the presence of corporate hypocrisy. We therefore formulate the following hypothesis:

H3: Stakeholder-driven motivation is positively associated with corporate hypocrisy.

### Corporate CSR Communication

To a certain extent, the intensity of communication relating to an enterprise’s CSR behavior reflects the enterprise’s underlying CSR motivation. Internally motivated (value-motivated) CSR behavior does not inherently require frequent communication. If an enterprise communicates its CSR activities too intensively, this may cause consumers to question the enterprise’s motivation for participating in such behavior. This will weaken the positive effect on consumer perceptions that the CSR behavior achieves. We formulate the following hypothesis:

H4: Value-driven motivation is negatively associated with corporate communication.

From the perspective of commercial interests, if the sincerity and sense of moral responsibility of an enterprise are not recognized by its stakeholders, their CSR behaviors may not yield any economic benefit (Fassin and Buelens, 2011). Therefore, many enterprises have invested a lot of energy in the communication of their own CSR behavior, trying to position themselves as enterprises with social responsibility. We formulate the following hypothesis:

H5: Performance-driven motivation is positively associated with corporate communication.

Some companies are not very keen to communicate their CSR behavior, but if some negative information is exposed affecting their CSR image, they will take measures to communicate their positive CSR behavior (Murray and Vogel, 1997). In general, a passive CSR communication strategy of this kind has the characteristics of using CSR communication to solve problems. Enterprises often use CSR communication to deal with unexpected phenomena in the market (Mou et al., 2012). After the occurrence of a problem, and in response to pressure from some stakeholders, the enterprise is likely to increase the intensity of its communication regarding its CSR behavior in order to protect or repair the image of the enterprise. We formulate the following hypothesis:

H6: Stakeholder-driven motivation is positively associated with corporate communication.

### CSR Implementation

Different enterprises exercise different levels of intensity in implementing CSR behavior. In some enterprises, CSR plays a very important role in enterprise management. Managers give CSR the same importance as quality management, human resources, and public relations. In other companies, CSR behavior is given a secondary level of importance, and such behavior is undertaken only due to the requirements of external rules and systems (Chaudhri, 2006). In some companies, CSR is regarded as a strategic behavior; in others, it is regarded as a forced cost (Mou et al., 2012).

Value-driven social responsibility means that enterprises have a moral motivation to seek to improve social welfare; that is to say, enterprises not only play an economic role in society but also create social value for society. In other words, the value-driven motivation for CSR behavior comes from the pursuit of social value creation. Social responsibility means that enterprises take action to ensure that decisions and behaviors are undertaken based on the interests of society, focus on reviewing and improving their production and operation from the perspectives of stakeholders and society in general, and effectively manage the impacts of their operation on society, stakeholders, and the environment. Therefore, we formulate the following hypothesis:

H7: Value-driven motivation is positively associated with CSR implementation.

The central motivation for engaging in corporate hypocrisy comes from the pursuit of purely economic interests, which means that the enterprise adheres to its commercial interests and regards shareholders’ interests as the central motivating factor in corporate development. Moreover, corporate hypocrisy reflects the fact that enterprises are motivated to make use of principles of “social responsibility” and the value assigned to ethical considerations by the public in pursuit of their commercial objectives.

Enterprises with performance-driven CSR motivation are not genuinely motivated by the maximization of social welfare and use the creation of financial value for the enterprise as the standard against which to judge the effectiveness of enterprise actions. There are three possible courses of action available to such enterprises: one is to undertake no socially responsible actions and to do nothing to promote social welfare, that is to say, to commit the “sin of omission”; the next is to act in a manner that is contrary to principles of social responsibility, which is manifested as behavior with a lack of social responsibility; the third is to take actions that are perceived to be socially responsible, but with the underlying objective of the pursuit of private interests (Xiao et al., 2013). We formulate the following hypothesis:

H8: Performance-driven motivation is negatively associated with CSR implementation.

Stakeholder-driven CSR behavior can be regarded as an adaptive strategy used by enterprises in the current global environment of sustainable development. The concept of sustainable development is becoming increasingly popular, which is leading to changes in value orientation. That is to say, social responsibility has become a new behavioral requirement in modern society. This new value orientation is increasingly penetrating into the fields of investment and consumption, which is leading to the unprecedented development of socially responsible investment and sustainable consumption. At the same time, various social responsibility movements, such as the environmental protection movement, the human rights movement, and the production code movement, have been emerging and becoming widespread, and society expects enterprises to play a greater role in solving social problems (Xiao et al., 2013). It is in the context of these environmental changes that enterprises make adaptive strategic adjustments and respond to the pressure applied to them by the social responsibility movement (Mou et al., 2012). In this regard, we formulate the following hypothesis:

H9: Stakeholder-driven motivation is negatively associated with CSR implementation.

Social responsibility requires enterprises to ensure sufficient transparency, to objectively and impartially disclose the impact of their own operation on society, stakeholders, and the natural environment, and to disclose, in a timely and accurate manner, information pertaining to the ways in which they create comprehensive economic, social, and environmental value for society and stakeholders. Social responsibility means that enterprises should communicate with society and stakeholders and that the intensity of communication should match their actions. Enterprises should make CSR commitments derived from internal incentives and mainly moral considerations, should adhere to the principle of balance, and should disclose both the beneficial and adverse effects of their actions. In contrast, an obvious feature of corporate hypocrisy is that the enterprise aims to establish an image of social responsibility with the public, potentially exaggerating or even fabricating the positive significance of their actions. Corporate hypocrisy may mean that the intensity of CSR communication by enterprises is higher than the intensity of their CSR behaviors (Xiao et al., 2013). We therefore formulate the following hypothesis:

H10: CSR communication is positively associated with CSR implementation.

### Corporate Hypocrisy

“Corporate hypocrisy” refers to the phenomenon that the CSR concept propagated by enterprises is inconsistent with their actual actions (Wagner et al., 2009) in the process of undertaking social responsibility. “Intra-enterprise hypocrisy” refers to the fact that the beliefs, values, and standards held and claimed by the managers and general members of the organization are different from the actual actions; that is, “the words and deeds of the managers and general members of the organization are different” (Argyris and Schon, 1974).

As a concept easily confused with corporate hypocrisy, workplace bullying is defined as one or more individual repeatedly engaging in deliberate behavior that causes the bullied to feel humiliation, pain, and helplessness and endangers their working performance, damaging both individual staff members and the whole organization (Einarsen, 1999).

It can be seen that corporate hypocrisy is the behavior of enterprises that involves cheating and misleading stakeholders, such as consumers, while bullying behavior in the workplace is the deliberate harm done by the bully to the bullied (Feng, 2016). The purpose of corporate hypocrisy is to seek personal gain or deal with pressure by deceiving or misleading consumers (Skarmeas and Leonidou, 2013). Enterprises may conceal their own environmental pollution, quality, and safety problems and exaggerate or fabricate their CSR behaviors. The perpetrator of bullying may also not acknowledge his bullying behavior, much less report it (Becher and Visovsky, 2012; Moore et al., 2013).

Both corporate hypocrisy and workplace bullying need to be made public or exposed because if stakeholders such as the media or consumers fail to expose or discover corporate hypocrisy, companies will take chances and willfully damage the environment or the interests of other stakeholders. If bullying behavior in the workplace is not publicized or the bullied avoids responding, the bully will regard the silence and inaction of the bullied as an endorsement of his behavioral style, which indirectly leads to the recurrence of bullying behavior (Feng, 2016).

From the perspective of consequences, hypocrisy in an enterprise will lead to the perception of inconsistency between the words and deeds of its members, which will affect their job satisfaction, attendance rate, organizational commitment, and turnover intention (Philippe and Koehler, 2004). Bullying, meanwhile, causes frustration and reduces self-efficacy in the bullied (Einarsen et al., 2007), and, if they cannot escape from the bullying for a long time, can give them a strong sense of helplessness and work alienation; in the long term, this will lower their self-esteem level and productivity and harm team cohesion (Jin, 2018). The research of Ferris et al. (2008) shows that workplace bullying can negatively affect employees’ interpersonal behaviors, reduce job involvement, and thus impair job performance. This study focuses on the impact of CSR motivation perception on corporate hypocrisy.

Corporate hypocrisy refers to the gap between what companies say and what they do (Wagner et al., 2009). The level of hypocrisy is often measured as the gap between the firm’s commitments and actual behavior (Mou et al., 2012). Corporate communication of CSR behavior will enhance consumers’ cognition of CSR behavior but will also increase consumers’ suspicion regarding the motivation for that behavior (Marquis and Lounsbury, 2007). Because the enterprise’s communication will strengthen consumers’ expectations of CSR, if the enterprise’s CSR behavior does not meet these expectations, the disparity between the enterprise’s behavior and expectations will give rise to consumers’ perceptions of corporate hypocrisy. Therefore, the CSR communication strategy of an enterprise actually contains two aspects: those concerning profitability and morality. The mismatch between the two aspects is the biggest obstacle for an enterprise in the implementation of a CSR strategy (Attarça, 2005). An enterprise’s optimal CSR strategy is to find the best combination between its implementation strategy and the communication strategy in order to minimize consumers’ perceptions of corporate hypocrisy (Mou et al., 2012). We formulate the following hypothesis:

H11: CSR communication is positively associated with corporate hypocrisy.

Hypocritical enterprises try to establish a “responsible” image in the eyes of stakeholders and the public via the implementation of pseudo-social responsibility behaviors. This implicitly shows that public perception of a good reputation for social responsibility can bring substantial benefits to the enterprise, that is, the “reputation effect” of CSR behaviors (Xiao et al., 2013). However, hypocritical enterprises only appear or pretend to be “socially responsible,” whereas they are not willing to perform the necessary actions or may even behave in a contrary manner. This shows that enterprises implicitly recognize that socially responsible actions come with costs, including the need to invest in tangible resources, intangible resources, organizational capabilities, and other enterprise resources. It can be seen that although enterprises that behave in accordance with pseudo-social responsibilities believe that the “reputation effect” of CSR can bring commercial benefits to enterprises, such enterprises perceive social responsibility as a burden and show that they are not willing to incur the cost of this responsibility to obtain the associated benefits (Xiao et al., 2013). We formulate the following hypothesis:

H12: CSR implementation is negatively associated with corporate hypocrisy.

## Methods

**FIGURE 1:**
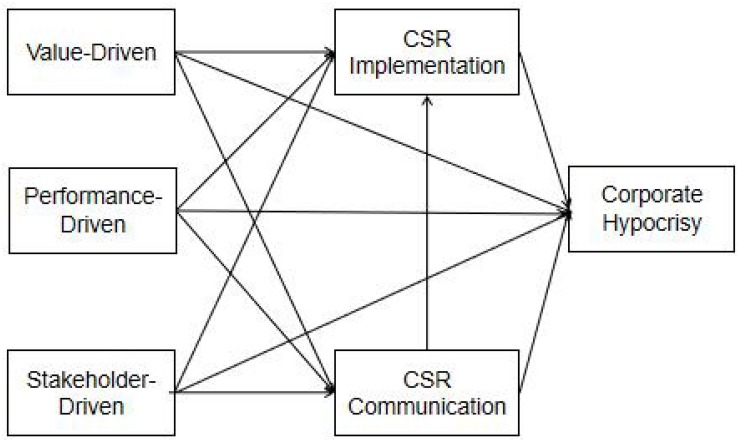
Research framework.

SmartPLS, which is suitable for small-sample data processing, deals with complex phenomena by means of statistics and analysis and research into the relationships between various variables. Perceptions and motivations of CSR and perception of hypocrisy are subjective factors that are difficult to measure directly, so we need to use some such variables and indicators for indirect measurement and description. This allows CSR motivation perception and corporate hypocrisy perception to be evaluated, and smartPLS can be used to construct a model of CSR motivation perception and corporate hypocrisy perception evaluation and to measure the degree of consumers’ perception of different CSR motivations and corporate hypocrisy. Based on this, this paper constructs a model (shown in Figure 1) through smartPLS to measure the relationship between CSR motivation perception, CSR communication, and CSR implementation as independent variables, as well as the relationship between CSR implementation and communication and consumers’ perception of corporate hypocrisy.

### Research Objects

According to the research of Maignan and Ralston (2002) and Ma et al. (2014), CSR communication and CSR implementation measurement require an open CSR social responsibility report. The specific criteria for sample screening are: ➀ The company is a listed company; ➁ The enterprise provides an open CSR report. According to the above selection principles, this study selected 17 enterprises from the Southern Weekend “Greenwashing List” and 11 enterprises participating in Wang Yangming’s conscience study. A total of 28 enterprises were included as research samples.

Since real motivation is embedded in the decision-making process of enterprises and cannot be directly measured and evaluated, this paper attempts to study the customer perception and evaluation of CSR activities from the perspective of public perception and attribution. Another reason for choosing this perspective is that the final effect of CSR activities depends not on the social responsibility activities themselves but on consumers’ perception, attribution, and subsequent behavioral responses, such as consumer trust and consumer support, and even the reward or punishment effect of the capital market determined by the investment behavior of the public.

Based on this, this study selected a total of 84 undergraduates and postgraduates majoring in management to participate in a questionnaire survey, including 43 women and 41 men. Each participant was asked to read and analyze the social responsibility report of the enterprise for the period from 2016 to 2018 and the news materials given and then to complete a questionnaire on the enterprise for 2016–2018.

### Measurement of Variables

The questionnaire items were translated into Chinese from English by professional institutions, and further translated and revised with three professors and two doctoral students of psychology and business management. A preliminary survey was conducted on a group of 31 people, and the questionnaire items were revised and improved according to the interview and preliminary survey results. The items of all factors are shown in Supplementary Table S1 in the Supplementary Appendix.

#### Independent Variables

CSR motivation perception. Following the approach developed by Maignan and Ralston (2002), we measure CSR communication based on corporate websites, annual reports, other public documents, and news sources. We conducted a comprehensive search of all the above resources covering the period from January 2017 to August 2019. In order to ensure consistency in the coding data, the data analysis of the annual reports, CSR reports, and company web pages was conducted by only one researcher, and their work was verified by others. According to Maignan and Ralston (2002), three types of CSR can be identified: (1) motivation of CSR activities; (2) managerial CSR processes; and (3) stakeholder issues. Based on the research of Du et al. (2007), Romani and Grappi (2014), and Lattemann et al. (2009), the present study codes the motivation of CSR implementation based on allocation into one of three different categories: (1) value-driven, (2) performance-driven, or (3) stakeholder-driven.

CSR communication, drawing on the research of Maignan and Ralston (2002), including the management of CSR process and stakeholder issues, as shown in Supplementary Tables S2, S3 in the Supplementary Appendix. We measure CSR communication based on corporate websites, annual reports, other public documents, and news sources. From January 2017 to August 2019, we conducted a comprehensive search of all the above resources. In order to ensure consistency in the coding data, the data analysis of documents is conducted by only one researcher, and the work verified by others. There are 21 items in the measurement scale. If the documents of one enterprise involve a certain item, it will get one point; otherwise, it will get no point. CSR communication ranges from 0 to 21.

CSR implementation. The method of measurement of the CSR implementation variables is based on the method used by Ma et al. (2014), and the items are shown in Supplementary Table S4 in the Supplementary Appendix. The intensity of CSR implementation is quantified by measuring the total number of times the enterprise has implemented CSR behaviors aimed at different stakeholders (shareholders, creditors, employees, customers, suppliers, society, the environment, and government). The greater the number of CSR behaviors are implemented for stakeholders, the stronger the CSR implementation is judged to be. The types of stakeholders involved represent the breadth of CSR implementation.

#### Dependent Variable

The measurement of corporate hypocrisy is based on Wagner et al. (2009). We used seven-point Likert-type scales from 1 = “completely disagree” to 7 = “completely agree” to complete statements starting, “In my opinion…,” as follows. (1) The enterprise acts hypocritically. (2) What the enterprise says and does are two different things. (3) The enterprise pretends to be something that it is not. (4) The enterprise does exactly what it says.^*v*^ (5) The enterprise keeps its promises.^*v*^ (6) The enterprise puts its words into action.^*v*^ (^*v*^ means reverse code).

## Results

In order to test the consistency and stability of the items, the external loading will be used for a reliability test. According to judgment based on experience, the external loading of each item should be greater than 0.4, and the external loading of all items measuring the same variable should be greater than 0.80. It can be seen from Table 1 that the external loading of each item is between 0.824 and 0.979, indicating that the reliability is sufficient (Hair et al., 2017).

**TABLE 1:** Item loadings.

	**CC**	**CH**	**CI**	**PD**	**SD**	**VD**
D1						0.966
D2						0.97
D3						0.911
J2				0.914		
J3				0.906		
S1					0.961	
S2					0.971	
G2	0.972					
G4	0.966					
X1			0.899			
X2			0.881			
Y1		0.952				
Y2		0.963				
Y4		0.979				
Y5		0.978				
Y6		0.824				
						

Cronbach’s alpha and AVE are used to test the validity of all latent variables. The Cronbach’s alpha coefficient of all items of the same variable should be greater than 0.70, and 0.5 is the acceptable critical value of AVE (Fornell and Larcker, 1981). It can be seen from Table 2 that Cronbach’s alpha of each variable is greater than 0.7, ranging between 0.739 and 0.967, and that the AVE of each variable is greater than 0.5, ranging between 0.793 and 0.939, indicating that the validity is sufficient.

**TABLE 2:** Cronbach’s alpha and AVE.

	**Cronbach’s Alpha**	**rho_A**	**C.R**	**AVE**
CC	0.935	0.942	0.969	0.939
CH	0.967	0.973	0.975	0.886
CI	0.739	0.742	0.884	0.793
PD	0.792	0.794	0.906	0.828
SD	0.93	0.945	0.966	0.934
VD	0.945	0.963	0.965	0.901

Tables 3, 4 show that the discriminant validities of the Fornell-Larcker criterion and HTMT test variables are within the acceptable range, mostly lower than the threshold of 0.90 (Henseler et al., 2014).

**TABLE 3:** Discriminant validity: Fornell-Larcker criterion.

	**CC**	**CH**	**CI**	**PD**	**SD**	**VD**
CC	0.969					
CH	0.241	0.941				
CI	0.807	0.377	0.89			
PD	0.475	0.071	0.409	0.91		
SD	0.422	0.149	0.357	0.911	0.966	
VD	−0.039	−0.818	−0.255	0.202	0.136	0.949

**TABLE 4:** Discriminant validity: Heterotrait-Monotrait ratio (HTMT).

	**CC**	**CH**	**CI**	**PD**	**SD**	**VD**
CC						
CH	0.249					
CI	0.966	0.442				
PD	0.549	0.121	0.534			
SD	0.447	0.156	0.428	1.055		
VD	0.079	0.848	0.3	0.248	0.156	

**FIGURE 2:**
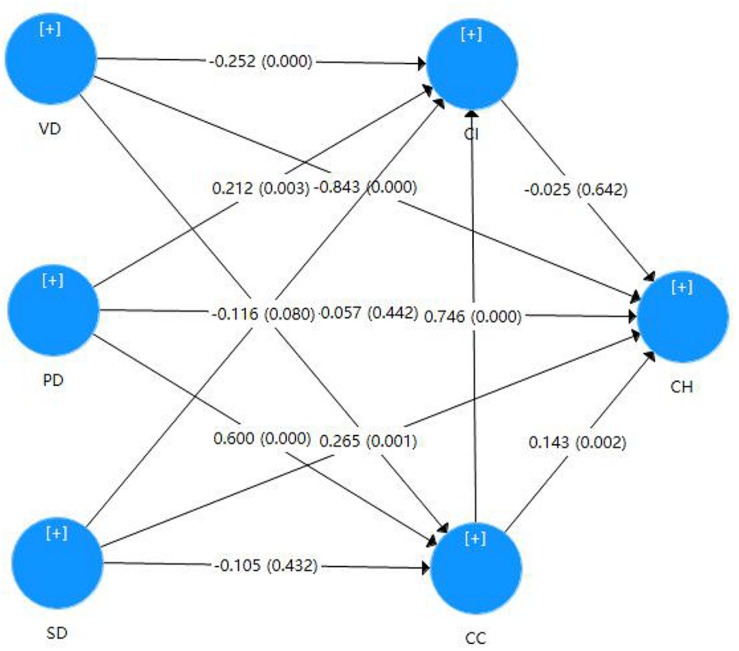
Resultsof the model.

As shown in Figure 2 and Table 5, H1 predicts that there is a negative correlation between value-driven motivation and corporate hypocrisy. In support of H1, the results showed that the higher consumers’ perception of value-driven motivation is, the lower their perception of corporate hypocrisy (the regression coefficient was -0.843, *P* < 0.001). When consumers attribute CSR activities to value-driven motivations, they may believe that the enterprise desires to solve social problems and improve overall social welfare. This may cause consumers to give respect and recognition to the enterprise and may reduce suspicion of corporate hypocrisy.

**TABLE 5:** Hypothesis testing results.

**Hypothesis**	**Original SAmple (O)**	**Sample Mean (M)**	**Std Dev. (STDEV)**	***T*-value (| O/STDEV|)**	***P*-value**	**Result**
H1 = VD - > CH	–0.843	–0.839	0.032	26.531	0	Supported
H2 = PD - > CH	–0.057	–0.065	0.074	0.78	0.436	Not supported
H3 = SD - > CH	0.265	0.272	0.08	3.32	0.001	Supported
H4 = VD - > CC	–0.147	–0.153	0.056	2.603	0.01	Supported
H5 = PD - > CC	0.6	0.596	0.12	4.995	0	Supported
H6 = SD - > CC	–0.105	–0.1	0.123	0.85	0.396	Not supported
H7 = VD - > CI	–0.252	–0.254	0.031	8.149	0	Not supported
H8 = PD - > CI	0.212	0.209	0.066	3.214	0.001	Not supported
H9 = SD - > CI	–0.116	–0.116	0.06	1.945	0.052	Not supported
H10 = CC - > CI	0.746	0.747	0.03	24.567	0	Supported
H11 = CC - > CH	0.143	0.14	0.047	3.069	0.002	Supported
H12 = CI - > CH	–0.025	–0.02	0.051	0.486	0.627	Not supported

H3 predicts that stakeholder-driven motivation is positively correlated with corporate hypocrisy. In support of H3, the results show that the higher the consumer’s perception of stakeholder-driven motivation is, the higher their perception of corporate hypocrisy (the regression coefficient is 0.265, *P* < 0.01). When consumers attribute CSR activities to stakeholder-driven motivations, they may believe that the enterprise’s underlying objective for such activities is to respond to pressure from stakeholders, thus increasing suspicion of corporate hypocrisy (Mou et al., 2012).

H4 predicts that value-driven motivation is negatively correlated with corporate communication. In support of H4, the results show that the higher the consumer perception of value-driven motivation is, the lower the amount of CSR communication (the regression coefficient is -0.147, *P* < 0.01). Value-driven enterprises, in order to meet their inner moral needs, do not need frequent publicity.

H5 predicts that performance-driven motivation is positively correlated with corporate communication. In support of H5, the results show that the higher the consumer perception of performance-driven motivation is, the higher the amount of CSR communication (the regression coefficient is 0.6, *P* < 0.001). When CSR activities are recognized by consumers, an image as a socially responsible enterprise can be formed, and thus performance benefits can be obtained.

Hypothesis 10 predicts that there is a positive correlation between CSR communication and CSR implementation. In support of hypothesis 10, the results indicate that the higher the amount of corporate CSR communication is, the higher the CSR implementation (regression coefficient 0.746, *P* < 0.001). Hypothesis 11 predicts that there is a positive relationship between CSR communication and corporate hypocrisy. In support of hypothesis 11, the results indicate that the higher the amount of CSR communication is, the higher corporate hypocrisy that consumers perceive (the regression coefficient is 0.143, *P* < 0.01).

The consumer perception of corporate hypocrisy is determined by the difference between the degree of effort put into CSR activities by the enterprise and the degree of effort that the enterprise claims to have made in its communications. Effective implementation of CSR behavior and appropriate CSR communication will bring positive evaluations of the enterprise from the public and avoid perceptions of corporate hypocrisy. Therefore, enterprises’ CSR implementation strategy and communication strategy should influence each other and be treated as interdependent in corporate strategy. If the CSR implementation of an enterprise falls short of the expectations set by its CSR communication, and this gap is large, it is easy to arouse consumers’ suspicion of corporate hypocrisy.

H2, H6, H7, H8, H9, and H12 are all rejected, and the results for hypotheses 9 and 12 are not significant.

H8 predicts that performance-driven motivation is negatively correlated with CSR implementation. This is rejected, as the results show that the higher the consumer perception of performance-driven motivation is, the higher the CSR implementation (the regression coefficient is 0.212, *P* < 0.001). The reason for the positive correlation between performance-driven motivation and CSR implementation may be that performance-driven enterprises regard CSR behavior as a tool. The greater the amount of CSR implementation, the better the image of the enterprise in the minds of consumers, and the higher the performance of the enterprise.

## Theoretical and Managerial Implications

### Theoretical Implications

To find out to what extent CSR communication drives CSR implementation, how they influence corporate hypocrisy, and what the key mechanisms are, we quantitatively investigated the key variables and their correlations. According to our findings, CSR communication does affect CSR implementation positively, but neither the former nor the latter variable significantly impact perception of corporate hypocrisy directly. Instead, the motivations driving CSR activities play substantial roles. On the one hand, they affect the intensity of CSR communication and engagement in CSR implementation contrarily; on the other, they significantly impact the perception of corporate hypocrisy. This is a remarkable finding. It implies that although consumers perceive corporate hypocrisy based on the CSR communication and implementation of enterprises, their perception is ultimately determined by the perception of the firms’ motivations for CSR activities. In other words, CSR motivations are the most crucial factor in determining corporate hypocrisy perception.

Specifically, companies sincerely concerned about CSR issues and wishing to create more social or environmental besides commercial value (having value-driven motivation) tend to conduct more substantial CSR activities (CSR implementation), whereas firms that are more interested in their overall economic performance (having performance-driven motivation) or more reactive to pressures from stakeholders (having stakeholder-driven motivation) are likely to publicize their CSR concerns and efforts (CSR communication). Though consumers perceive corporate hypocrisy somewhat based on an enterprise’s CSR communication and implementation, they seemingly tend to probe the motivations of CSR activities to assess the company’s sincerity. According to our findings, if consumers perceive a value-driven motivation behind the firm’s CSR communication or implementation, they will assume that the firm is sincere in their CSR activities, and the disparity between words and deeds on CSR will be modest, thus developing a lower level of corporate hypocrisy perception; if they recognize a higher level of performance- or stakeholder-driven motivation underlying these activities, they will suspect the company’s substantial CSR activities and are more likely to speculate that there is a disparity between its CSR communication and implementation, thus eventuating a higher level of corporate hypocrisy perception. This result echoes the findings of some other relevant studies (e.g., Mou et al., 2012).

The findings of this study largely complement the definition of and research on corporate hypocrisy. First, although corporate hypocrisy perception is pertinent to the gap between CSR communication and implementation, it can be determined by the motivations behind them. Second, CSR motivations considerably affect the intensity of CSR communication and engagement in CSR implementation and can be detected by consumers, who may resort to subjective assessment of the likelihood of a disparity between CSR words and deeds based on their own perceptions instead of observing and recording the disparity patiently in future. Third, the essence of corporate hypocrisy remains, but perceiving it can depend on either waiting and identifying the disparity between words and deeds or estimating its likelihood. In other words, consumers can choose another approach (relatively subjective) besides the conventional one (relatively objective) to perceive corporate hypocrisy. The subjective approach starts from CSR communication and implementation, tracing back to the motivations behind them.

For the insignificant results, such as the results that value-driven motivation for CSR does not significantly influence the intensity of CSR communication and that stakeholder-driven motivation for CSR does not significantly influence engagement in CSR implementation, there may be a more complex mechanism involved. On the one hand, value-driven and stakeholder-driven motivation respectively lead to different levels of sincerity in CSR and thus different attitudes toward CSR communication and implementation, separately; on the other hand, these motivations may respectively kindle more concern about the firms’ CSR efforts, which should not blindly increase or decrease CSR communication and implementation. This again supports our argument that, within a limited time frame, it might be rather challenging for consumers to identify corporate hypocrisy simply based on communication intensity and implementation engagement of CSR, triggering them to employ an additional mechanism to estimate the likelihood of a disparity between CSR communication and implementation. Revealing the details in the process from the value-driven motivation to CSR communication and from stakeholder-driven motivation to CSR implementation requires more in-depth research in future.

### Managerial Implications

These findings can help company managers to identify the CSR motivations of their firms and to speculate on their impacts on subsequent CSR communication and implementation as well as perceptions of consumers regarding corporate hypocrisy. Second, this study may help managers to become more aware of CSR implementation since their firm’s communication on CSR will eventually drive it toward substantial engagement, and the factual disparity between what they say and what they do will be identified sooner or later. Third, although consumers may adopt the relatively subjective approach (detecting CSR motivations) to develop their perception of corporate hypocrisy, they will have to do it based on the CSR communication and implementation observed. Since the aforementioned substantial engagement is inevitable, well-balanced resource allocation on each of these might not only help decrease the objective disparity between CSR words and deeds, but also the subjectively estimated likelihood of that disparity (consumers detect the firm’s motivations behind its communication and implementation).

## Limitations and Further Research

### Limitations

This study is not without limitations. First, it adopts a structural equation model as its major method, estimating the net correlation, but further investigation on causality and joint effects of the variables may require that other methods be employed, such as fuzzy qualitative comparative analysis. Second, this research implies that there might exist more complex mechanisms that decrease the direct impact of CSR communication and implementation on corporate hypocrisy perception. Thus, it is necessary to deploy in-depth qualitative approaches to reveal the complex mechanisms that might mutually interfere with consumers’ perception and judgment of corporate hypocrisy. Third, due to complementarity, efforts may be necessary to compare the results based on relatively objective estimation (of the disparity between CSR communication and implementation) and on subjective estimation (of the likelihood of a disparity between CSR communication and implementation), which involves longitudinal approaches.

### Further Research

More variables and a larger sample size involving demographic and regional differences are suggested for further research to facilitate the causal mechanism to be more comprehensively revealed and to enhance the external validity of this study.

## Data Availability Statement

The datasets generated for this study are available on request to the corresponding author.

## Author Contributions

YZ was responsible for the manuscript writing. YQ was responsible for the manuscript revision. XZ was responsible for the interview. XW was responsible for the theoretical framework and implications. LS was responsible for the methodology.

## Conflict of Interest

LS was employed by company State Grid Hebei Electric Power Company Xingtai Power Supply Branch. The remaining authors declare that the research was conducted in the absence of any commercial or financial relationships that could be construed as a potential conflict of interest.
