# Supplementary material for: Perception of Corporate Hypocrisy in China: The Roles of Corporate Social Responsibility Implementation and Communication
Source: Front Psychol. 2020 Apr 22;11:595. doi: 10.3389/fpsyg.2020.00595 (PMC7212433; doi:10.3389/fpsyg.2020.00595)
Supplement: Supplementary file 1 [file Data_Sheet_1.doc]

Appendix

Table S1 Variable description

| Factors | Symbol | Items |
| --- | --- | --- |
| Value-Driven | D1 | CSR is presented as being part of the company’s culture, or as an expression of its core values. |
|  | D2 | I think that ** feels morally obligated to help environment and society |
|  | D3 | I think that ** has a real, authentic long term interest in the environment |
| Performance-Driven | J1 | CSR is introduced as a part of the firm’s economic mission, as an instrument to improve its financial performance and competitive posture. |
|  | J2 | I think that ** is taking advantage of environmental causes to help its own business |
|  | J3 | I think that ** seeks actually to get publicity |
| Stakeholder-Driven | S1 | CSR is presented as a response to the pressures and scrutiny of one or more stakeholder groups. |
|  | S2 | I think that ** is taking advantage of environmental causes to help its own business |
|  | S3 | I think that ** seeks actually to get publicity |
| CSR communication | G1 | Processes of CSR (See Table 7 for details) |
|  | G2 | Stakeholder issues(See Table 7 Continued for details) |
|  | G3 | ** the intensity of CSR communication is very high. |
| CSR implementation | X1 | The breadth of implementation (See Table 8 for details) |
|  | X2 | The strength of implementation (See Table 8 for details) |
|  | X3 | ** the intensity of CSR implementation is very high. |
| Corporate hypocrisy | Y1 | ** acts hypocritically. |
|  | Y2 | What ** says and does are two different things. |
|  | Y3 | **pretends to be something that it is not. |
|  | Y4 | **does exactly what it says.^v^ |
|  | Y5 | **keeps its promises.^v^ |
|  | Y6 | **puts its words into action.^v^ |

Note:(v item mean reverse code)

Table S2 CSR communication scale(Maignan and Ralston, 2002)

| Processes of CSR |  | Philanthropic programs | The company presents a formalized philanthropy program made with a clear mission and application procedures to allocate donations and grants. |
| --- | --- | --- | --- |
|  |  | Sponsorships | The company introduces sponsorships as a type of responsibility initiative aimed at providing assistance, either financial or in-kind, to a cause or charity. |
|  |  | Volunteerism | The company presents programs that allow employees to work for a good cause during paid working hours. |
|  |  | Code of ethics | The company discusses the content and/or implementation of a code of ethics or conduct. |
|  |  | Quality programs | The company describes a formal product/service quality program as a form of responsibility initiative. |
|  |  | Health and safety programs | The company introduces formal health and safety programs aimed at one or more stakeholder groups as a form of responsibility initiative. |
|  |  | Management of environmental impacts | The company discusses activities aimed at diminishing the negative impact of productive activities on the natural environment. |

**Table S3 Continued CSR communication scale(Maignan and Ralston, 2002)**

| Stakeholder issues | Community Stakeholders | Arts and culture | The company discusses its support of organizations, activities, actors, and objects linked to the arts or the national culture. |
| --- | --- | --- | --- |
|  |  | Education | The company presents its support of activities aimed at improving educational opportunities and the quality of education received by populations outside of the ﬁrm. |
|  |  | Quality of life | The company expresses its dedication to improving the quality of life and the well-being of the communities in which the ﬁrm operates, or the society as a whole. |
|  |  | Safety | The company displays concern for the safety of the persons in the communities surrounding its productive operations. |
|  |  | precision poverty relief |  |
|  |  | Protection of the environment | The company shows concern for the preservation of the natural environment,either in general or in the communities where the ﬁrm operates. |
|  | Customer Stakeholders | Quality | The company presents achievement of high product/service quality as a part of its commitment to social responsibility. |
|  |  | Safety | The company displays concern for the safety of its customers in relation to its production activities or products/services. |
|  | Employee Stakeholders | Equal opportunity | The company expresses its commitment to giving the same recruitment and promotion chances to all employees regardless of race, gender, age, or disability. |
|  |  | Health and safety | The company expresses its concern for protecting the safety of employees in the workplace as well as for the level of their overall health. |
|  | Shareholders |  | The company expresses its commitment to the involvement of stakeholders incorporate governance and/or support for providing stakeholders with proper information. |
|  | Suppliers |  | The company expresses its dedication to giving equal opportunities to suppliers in terms of gender, race, and size and/or assuring suppliers’ safety. |
|  |  |  | Formulate standardized procurement management system, strengthen supplier management and business cooperation, and protect the legitimate rights and interests of suppliers. |
|  |  |  | Establish upstream and downstream supply chain management to ensure common development of partners |

Table S4 CSR Implementation Scale

| CSR Implementation | The breadth of implementation | CSR Implementation to shareholders |
| --- | --- | --- |
|  |  | CSR Implementation to employees |
|  |  | CSR Implementation to customers |
|  |  | CSR Implementation to suppliers |
|  |  | CSR Implementation to society |
|  |  | CSR Implementation to environment |
|  |  | CSR Implementation to government |
|  | The strength of implementation | Number of CSR Implementation actions to shareholders |
|  |  | Number of CSR Implementation actions to employees |
|  |  | Number of CSR Implementation actions to customers |
|  |  | Number of CSR Implementation actions to suppliers |
|  |  | Number of CSR Implementation actions to society |
|  |  | Number of CSR Implementation actions to environment |
|  |  | Number of CSR Implementation actions to government |
